# Fabrication of superhydrophobic composite coating based on fluorosilicone resin and silica nanoparticles

**DOI:** 10.1098/rsos.180598

**Published:** 2018-07-25

**Authors:** Xiaoming Wang, Xingeng Li, Qingquan Lei, Yaping Wu, Wenjing Li

**Affiliations:** 1 Material Laboratory of State Grid Corporation of China (Shandong), State Grid Shandong Electric Power Research Institute, Jinan 250001, People's Republic of China; 2 College of Material, Science and Engineering, Qingdao University of Science and Technology, Qingdao 266042, People's Republic of China

**Keywords:** composite coating, superhydrophobic, fluorosilicone resin

## Abstract

Composite superhydrophobic coating built with film former and filler is attracting much attention for its facile and convenient fabrication, but significant limitations and disadvantages still remain. In this paper, a composite superhydrophobic coating is introduced which can be cured at room temperature and made by dispersing modified silica nanoparticles with 1H, 1H, 2H, 2H-perfluorooctyltriethoxysilane in fluorosilicone resin. Silica content and dispersion time showed obvious influences on the morphology and topography of composite coating by reuniting dispersed nanoparticles to form peaks on the surface. Excessively large distances between these peaks would decrease water contact angle value. Increasing slope of peaks, appropriate distance between peaks and decreasing diameter size of peaks would diminish sliding angle value. Formation mechanism of the composite coating based on fluorosilicone resin and modified nanoparticles was explained using interpenetrating polymer model.

## 1. Introduction

Superhydrophobic surfaces have been investigated for two decades [1,2] in many potential applications owing to their perfect self-cleaning and water proof ability resulting from their high contact angle (CA) and low sliding angle (SA) [3–8]. It is widely accepted that micro/nano-hierarchical structures and low surface energy components are the two key factors to form superhydrophobicity [9–12]. Superhydrophobic surfaces are generally fabricated through two strategies, they modify a rough surface with low surface energy materials and roughen low surface energy surface.

Composite superhydrophobic material is usually fabricated with film former and nanoparticles. Nanoparticles are used to form the micro/nano-hierarchical structure with hydrophobicity through modification [13,14]. Cross-linked polymers are chosen as a film former to bond nanoparticles, such as epoxy resin [15–19], polyurethane [15,20,21], acrylic resin [22,23], polystyrene [23,24], phenylene sulfide [25,26], polydimethylsiloxane [27], polyvinylchloride [5] and silicon rubber [28]. Though many superhydrophobic coatings have been successfully obtained by this composite method, significant limitations and disadvantages still exist. For example, ultraviolet (UV) or high-temperature curing is required, which inhibits its wide application. Weak ageing resistance and high surface energy or hydrophilicity of some polymers made superhydrophobic coatings have a short service life. Sometimes, low surface energy material was selected within composite material to reduce surface energy of composite coating. However, these additives have a short life and high price. As a result, there is a strong need to develop a kind of long life, room temperature curing and low surface energy composite superhydrophobic coating.

Fluorosilicone resin is a combined material with fluorinated groups and siloxane compositions which have room temperature curing, good weatherability and low surface energy [29,30]. These advantages make it a suitable candidate in manufacturing superhydrophobic materials [30–32]. Superhydrophobic coatings fabricated with fluorosilicone resin, nanoparticles and a large mass ratio of epoxy resin have been reported [33,34], but the performance of these coatings was not favourable owing to the poor UV resistance and high surface energy of epoxy resin. There have been few studies done on fabricating superhydrophobic coating just with fluorosilicone resin and nanoparticles because of weak linkage between fluorosilicone resin and nanoparticles.

The aim of this paper is to fabricate superhydrophobic coating only based on inert fluorosilicone resin and modified nanoparticles. The effect of process and filler contents on structure and wettability of composite coating has been investigated. The relationship between structure character and wettability of the coating was analysed and evaluated. Moreover, a formation mechanism of superhydrophobic coating with preferred surface structure was built.

## 2. Experiment

### 2.1. Materials

Commercial glass plates were selected as substrates. Fluorosilicone resin (Dongfu Chemical Technology Co. Ltd) with more than 20% fluorine content was used in this experiment. The curing agent (HDI trimer) used in this study is commercial product (n3390) which was purchased from Bayer. Silica nanoparticles (Hongde Nanomaterials Co. Ltd) have an average size of about 60 nm. 1H, 1H, 2H, 2H-perfluorooctyltriethoxysilane (PTES) was purchased from Harbin XEOGIA Co. Ltd. Ethyl acetate, butyl acetate, absolute ethyl alcohol and acetic acid were in analytical grade and used as received without any further purification.

### 2.2. Sample preparation

#### 2.2.1. Preparation of modified silica nanoparticles

Ten grams of silica nanoparticles were dispersed in 100 g of absolute ethyl alcohol under high shearing emulsification at 6000 r.p.m. for 10 min and ultrasonic for 10 min, respectively. After that, the emulsion was stirred at 1400 r.p.m. with a magnetic stirrer at 60°C. Five grams of PTES were added into the emulsion. At the same time, 50 g absolute ethyl alcohol and 5 g water were blended with a glass rod, and the pH value of the solution was adjusted to 3 with acetic acid. The solution was added to the emulsion by dripping slowly and followed by 2 h stirring. The silica particles were dried and washed repeatedly with a mixture of deionized water and ethanol three times. Modified silica particles were collected when dried.

#### 2.2.2. Preparation of composite coating

Firstly, we made the nanocomposite paint. Ethyl acetate was mixed with butyl acetate. Silica particles were dispersed in this solution with high-speed shearing and ultrasonic sonication at room temperature for 15 min, respectively. Fluorosilicone resin was added to this solution with mechanical stirring. Then, the nanocomposite paint was ready.

Secondly, superhydrophobic coating was fabricated. Glass substrates were cleaned by deionized water and dried by hot-blast air. HDI trimer was mixed in the nanocomposite paint with mechanical stirring for 5 min. The ratio of HDI trimer and fluorosilicone resin was 1 : 10. Finally, the glass substrates were sprayed with the paint by compressed air at a pressure of 0.3 MPa.

### 2.3. Sample characterization

Contact angle (CA) and sliding angle (SA) were carried out on OCA-20 (DataPhysics Corporation, Germany). The CA and SA values were the averages of three measurements by using 4 µl and 10 µl water droplets, respectively. The surface morphology and topography of the coatings were characterized by FESEM SUPRA 55 (ZEISS Corporation, Germany) and AFM Dimension Edge (Bruker, Germany). The chemical component was characterized by Fourier transformer infrared spectra (FTIR, Thermo Scientific Nicolet iS10). Differential scanning calorimetry (DSC) analysis was performed on a Dazhan DSC-100 L calorimeter. A heating rate of 20°C min^−1^ was used under a nitrogen flow of 20 ml min^−1^.

## 3. Results and discussion

### 3.1. Wettability of these coatings

**Figure 1.**
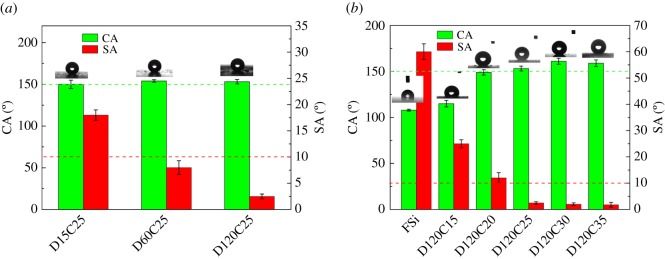
CA and SA of coatings with (*a*) three dispersion times, and (*b*) different silica content. The inserted graphics show the CA of coatings in (*a*) and (*b*).

All coatings are divided into two classes by dispersion process and filler content. D15C25, D60C25 and D120C25 are fabricated by stirring for 15, 60 and 120 min, respectively, with 25% silica nanoparticles. D120C15, D120C20, D120C25, D120C30 and D120C35 are fabricated by stirring for 120 min with 15, 20, 25, 30, 35% silica content, respectively. FSi is fluorosilicone resin coating. All the coatings are of a white appearance except that FSi is transparent and D120C15 is translucent. Surface wettability was investigated by CA and SA. Measurement results of these coatings are shown in figure 1.

The composite coatings with three dispersing times show similar CA value and are greater than 150°. D120C25 has the largest one, but the SA values of them are different. D15C25 has SA at 7.5°, the SA of D60C25 is at 4.6° and the SA of D120C25 is the smallest at 2.5°. All three coatings have superhydrophibicity with different SA. The chemical content of three coatings is the same. There must be some difference of surface structure in three coatings which made them different SAs. Silica nanoparticles were predispersed in solvent with the same process. The dispersion degrees of nanoparticles of three coatings are the same. The structure difference of three coatings is attributed to dispersion time of fluorosilicone resin in solution.

Silica content affects wettability of coatings too. FSi has 108° CA, which is close to that of polytetrafluoroethylene (PTFE) [35]. FSi coating is flat and the large CA reflects its low surface energy. D120C15 with 15% silica nanoparticles has a little larger CA than FSi. Silica particles are not enough to build effective rough structure on the coating surface with 15% content. CA of D120C20 is improved to about 150°, but SA is still larger than 10° which cannot be considered as superhydrophobic. When silica particles content is increased to 25%, CA of composite coating is 153° and SA is 2.5°. D120C30 and D120C35 with silica content more than 25% have little larger CA and similar SA. But these coatings are loose, and white particles could be peeled off these coatings. They cannot be used in practice. From these measurements and observations, it can be concluded that an outstanding superhydrophobic coating could be fabricated based on fluorosilicone resin with appropriate silica content and dispersion time.

### 3.2. Morphology of particles and coatings

**Figure 2.**
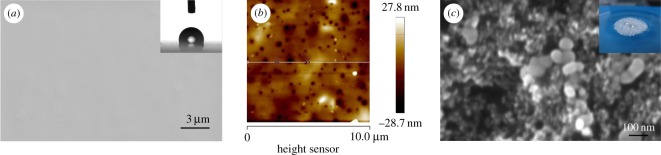
(*a*) SEM image of FSi resin. The inserted graphic shows the CA of FSi resin. (*b*) AFM image of FSi resin. (*c*) SEM image of modified silica nanoparticles. The inserted graphic shows the spherical water droplet on modified silica nanoparticles.

Scanning electron microscopy (SEM) was employed to display morphology of silica nanoparticles and fluorosilicone resin in figure 2. The surface of fluorosilicone resin was completely flat without any visible defect on it (figure 2*a*). It can be seen that the roughness of the resin coating is nanometre level from atomic force microscopy (AFM) image of fluorosilicone resin in figure 2*b*. The CA of it can reflect its surface energy. CA of fluorosilicone resin is shown in the upper right corner of figure 2*a*. The CA value is 108° mentioned in the previous section, and shows the perfect hydrophobicity of it.

SEM is used to measure size of silica particles in this paper. Modified silica nanoparticles were dispersed in absolute ethyl alcohol by shearing emulsification equipment at 9000 r.p.m. for 30 min and ultrasonic sonication for 15 min. The emulsion was dropped on conductive silver paste and dried. SEM images of the modified silica nanoparticles are shown in figure 2*c* with an average of 60 nm. These particles are spheres with uniform diameter size. The hydrophobicity of these silica particles was investigated by the water droplet method. Water seems to be spherical on the modified silica nanoparticles, which means the modified silica has favourable hydrophobicity.

Silica particles dispersion time influences hydrophobicity of composite coating as mentioned previously in figure 1, and SEM was used to reveal the different morphology of coatings with different dispersion time.

**Figure 3.**
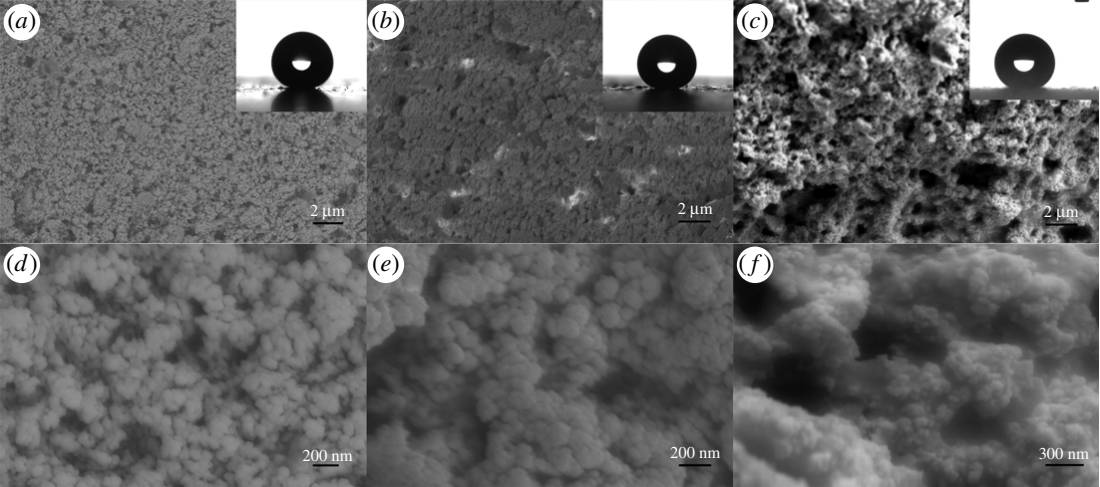
SEM images of (*a*) D15C25, (*b*) D60C25 and (*c*) D120C25. The inserted graphics of (*a–c*) show CA of these coatings. (*d–f*) Higher magnification of (*a–c*).

**Figure 4.**
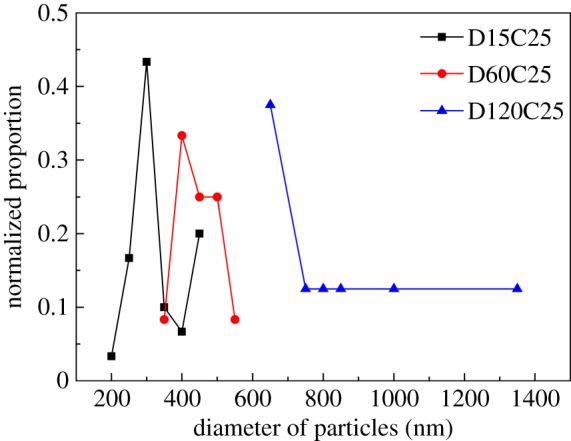
Distribution of diameter of particles of three coatings.

SEM images of three coatings with 15, 60 and 120 min dispersion time are shown in figure 3*a–c*. There are large submicron particles on the surface of D15C25. The size of these particles was uniform. D120C25 has the roughest surface with large number of irregular particles and pits, D60C25 has a surface in between. In magnified images figure 3*d–f*, differences of coatings seemed more obvious. The particle size statistics of these coatings are shown in figure 4. The particle size of D15C25 mainly distributes between 200–450 nm, while the particle sizes of D60C25 mainly distributes between 350–550 nm. The particle size of D120C25 is the largest and it distributes between 700–1350 nm. Silica nanoparticles could be distinguished in all three coatings, but submicron particles on D15C25 and D60C25 are smooth. This may be owing to fluorosilicone resin covering these particles. On D120C25 surface, silica nanoparticles could be seen clearly and they bonded compactly with each other by fluorosilicone resin. Fluorosilicone resin wetted silica nanoparticles well. It cannot be concluded whether fluorosilicone resin cover these nanoparticles or not, but these particles are distinct to those of D15C25 and D60C25. The roughness of D120C25 is larger than that of the other two coatings.

Three coatings have nearly the same CA and the images are displayed in the upper right of figure 3*a–c*. Water droplets are spherical on these coatings. All three coatings had large CA value because of the fluorosilicone layer and rough structure on these coatings. But SA of three coatings has great differences. The roughest structure of D120C25 does better on SA than that of the other two coatings. It can be said that rougher structure can improve SA values.

Filler particle is the key to forming rough surfaces of composite coatings. The content of particles has great impact on the surface structure of the composite coating. Coatings with different particle content were investigated here. All these composite coatings with modified silica particles were stirred for 2 h.

**Figure 5.**
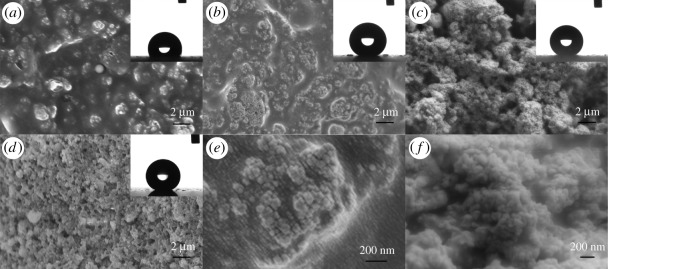
SEM images of coatings with (*a*) 15%, (*b*) 20%, (*c*) 25%, (*d*) 30% PTES silica nanoparticles, (*e*) higher magnification of (*b*), and (*f*) higher magnification of (*c*). The inserted graphics of (*a*–*d*) show CA of these coatings.

SEM images of these composite coatings with different content are shown in figure 5. Particles were seen and covered with fluorosilicone resin on D120C15 at 15% of silica nanoparticles. The size of these particles is from 1 to 20 µm. They are made from reuniting dispersed nanoparticles during the curing process. It is smooth on these particle surfaces, which means it is covered with fluorosilicone resin completely. Measurement of CA at 115° is shown in the upper right of figure 5*a*. The value of CA was slightly larger than that of fluorosilicone resin. These particles on the coating contribute to the increase in CA. The number of particles increase as the content of particles is up to 20% in figure 5*b*. There is a flat area with fluorosilicone resin between these particles. In the magnified image figure 5*e*, silica nanoparticles are identified and adhered to coating compactly. Fluorosilicone resin is infiltrated into the interval of these silica nanoparticles. Micro/nano-hierarchical structure is found on it. The CA image is spherical shown in the upper right corner of figure 5*b*. Though D120C20 has big CA at 149°, SA is at 12° for the flat area of the coating. The coating with content of 25% silica particles in figure 5*c* is the same with that in figure 3*c*. All silica nanoparticles are adhered to the surface compactly with fluorosilicone resin in figure 5*f*. Particles on the coating surface became loose when silica particles content is 30% in figure 5*d*. Silica nanoparticles can be seen clearly as in figure 5*f*. But particles on the coating could be peeled off easily for minute quantity of fluorosilicone resin between these particles. CA measured in the upper right corner of figure 5*d* is approximate with that in figure 5*c*. The loose structure on the coating give large CA values.

The observation from SEM images is that dispersion time and filler content are the cause of structure formation of composite coating based on fluorosilicone resin. The optimal composite coating of D120C25 has been selected with appropriate preparation process and filler content.

### 3.3. Film-forming property and wear resistance of coatings

**Figure 6.**
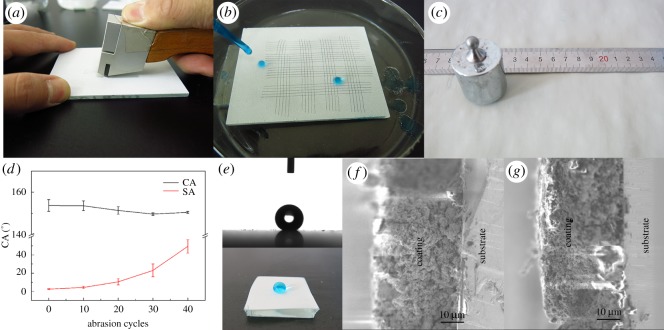
(*a*) Schematic illustration of the knife-scratch test. (*b*) Water droplet sliding off the knife-scratched composite coating. (*c*) Schematic illustration of sandpaper abrasion test. (*d*) Wear resistance measurement of the composite coating. (*e*) CA, photos of abraded composite coating. SEM images of cross-section of the composite coating (*f*) before and (*g*) after sandpaper abrasion test.

Cross-cut was processed to test film-forming property. D120C25 was selected to test. Schematic illustration of the cross-cut test based on standard ISO 2409 is shown in figure 6*a*. After the scratching, there are deep vertical cross-scratches on composite coating, and glass substrate can be seen in figure 6*b*. The result of the cross-cut test is class 1, and water droplets did not leave any mark when they rolled off the coating. The preferred composite coating has favourable film-forming property.

In this paper, wear resistance of the superhydrophobic surface was investigated via a sandpaper abrasion test [15,36]. D120C25 was selected to test. SiC sandpaper (1000 grit) was used to wear the surface. The coating surface with area of 2 cm × 2 cm is under a weight at 200 g. The applied pressure is about 8 kPa. The coating was abraded longitudinally and transversely for 10 cm, respectively, which is defined 1 cycle shown in figure 6*c*. CA and SA were measured every 10 abrasion cycles, and the result is shown in figure 6*d*. The coating maintained superhydrophobicity with CA larger than 150° and SA smaller than 10° after 20 cycles of abrasion. After 40 cycles, the CA was around 150°, while SA increased sharply to 49.5° in figure 6*e*. In the SEM image (figure 6*f*) of a cross-section of the composite coating before the abrasion test, the surface of it is coarse with many bulges. After the abrasion test, the surface becomes flat with nanoparticles on it as shown in figure 6*g*. It may be the reason why there is a sharp increase of SA with CA larger than 150°.

### 3.4. Chemical component of nanoparticle and coating

**Figure 7.**
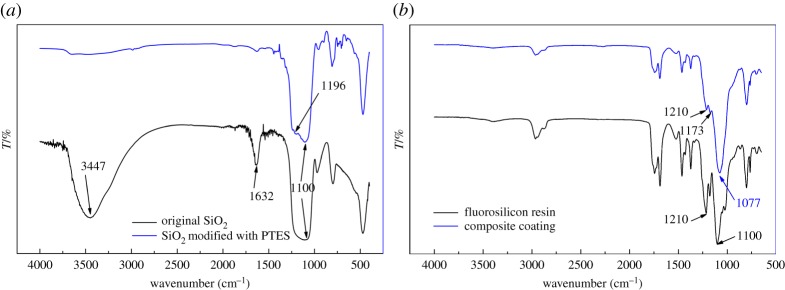
(*a*) FTIR spectra of untreated silica nanoparticles and those modified with PTES. (*b*) FTIR spectra of fluorosilicone resin and coating with silica particles modified with PTES.

It is known that silica is hydrophilic. Silica could become hydrophobic after being hydrophobicly modified. Silica nanoparticles used in this paper were modified with PTES. Chemical component is used to be confirmed by FTIR. In order to compare the difference between original and modified silica nanoparticles, the FTIR spectra of them are listed in figure 7*a*. From figure 7*a*, a broad absorption peak at 3447 cm^−1^ representing associating hydroxyls could be seen clearly in FTIR spectra of the original silica, while it was almost invisible in FTIR spectra of modified silica. The peak at 1632 cm^−1^ representing bending vibration of hydroxyls has been weakened in FTIR spectra of modified silica. Hydroxyls on silica were almost replaced completely. An adsorption peak at 1196 cm^−1^ appeared in FTIR spectra of modified silica. There were many small adsorption peaks in the finger-print region too. All these peaks represented fluorocarbon (F–C) bond. It could be concluded that the F–C bond came from PTES and silica nanoparticles were modified with FTES successfully. These modified silica nanoparticles have low surface energy for these F–C bonds on silica surface.

Chemical components of fluorosilicone resin and the composite coating are also investigated with FTIR. D120C25 is chosen to be the measured composite coating. FTIR spectra of D120C25 are shown in figure 7*b*. The peak at 1077 cm^−1^ is antisymmetric vibration of the Si–O–Si bond, which is clear in spectra of composite coating. The narrow adsorption peak at 1100 cm^−1^ of pure fluorosilicone resin disappeared in FTIR spectra of composite coating. It is close to and may be covered by the peak at 1077 cm^−1^. The narrow adsorption peak at 1210 cm^−1^ could be seen in both of the FTIR spectra. Both peaks at 1100 and 1210 cm^−1^ represent F–C bond. It can be concluded that composite coating contained F–C bond and composite coating has low surface energy, whether F–C bond is from PTFE or fluorosilicone resin.

### 3.5. Consideration of superhydrophobicity on structure of coatings

**Figure 8.**
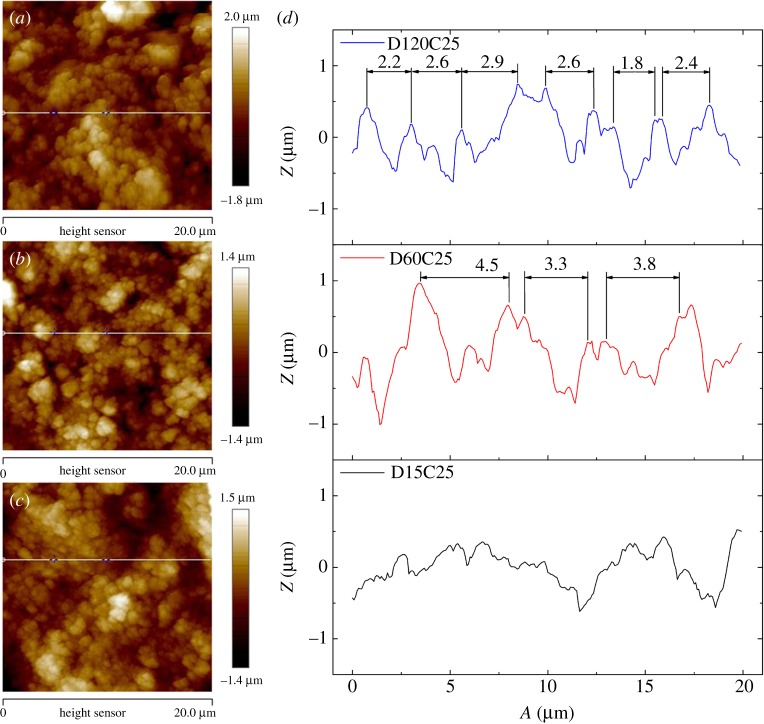
AFM images of (*a*) D16C25, (*b*) D60C25, (*c*) D120C25 and (*d*) roughness profile of (*a–c*).

**Figure 9.**
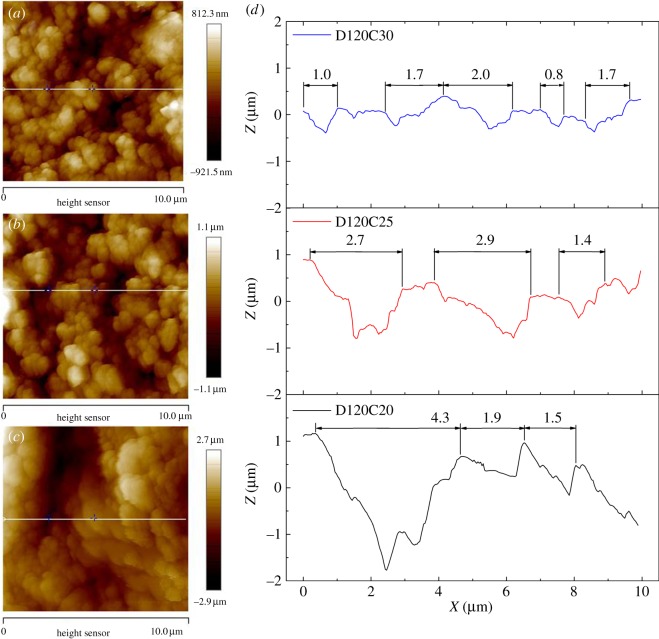
AFM images of (*a*) D120C20, (*b*) D120C25, (*c*) D120C30 and (*d*) roughness profile of (*a–c*).

It has been proved that composite coatings in this paper have low surface energy with the same chemical content. But the hydrophobicity of these coatings is different. Structure of composite coating is the main cause. Information from SEM images of composite coatings is not enough to decide the critical factors on superhydrophobicity. AFM images were detected and shown in figures 8 and 9.

The scan area of three coatings with different dispersion times is 20 × 20 µm². Figure 8*a–c* is the planar graph of them. Distribution laws of the three coating particles were totally different. D15C25 has large flat surfaces, while bulges of D60C25 and D120C25 are small pieces or bits. The character of three coatings could be seen more clearly in cross-section outlines shown in figure 8*d*. The D15C25 has many large bulges with very narrow valleys between of them. Very thin peaks are built on these bulge surfaces. When silica nanoparticles are dispersed for a longer time, large peaks and valleys with steep shapes are found on D120C25 and D60C25. But characters of these two coatings are different too. The number of peaks and distance between peaks are counted in figure 8*d*. D120C25 has more peaks and shorter distance than those of D60C25. Peaks slopes of D120C25 are bigger than those of D60C25 too (table 1). From figure 1*a*, close CA and different SA are observed. Different slopes and distances of these peaks effect CA little and SA remarkably.

**Table 1.** Peak slopes of D60C25 and D120C25.

coating		slope of peaks						
D120C25	right side	1.3	1.3	1.1	0.9	1.4	1.4	1.5
	left side	2.8	0.8	3.8	0.7	3.5	3.1	1.2
D60C15	right side	0.6	1.4	1.4	1.3			
	left side	2.0	1.9	1.9	1.1			

Figure 9 shows topography and the difference of coatings at different silica content. The scan area of these coatings is 10 × 10 µm². D120C20 has large bulges and valleys, while D120C25 and D120C30 have small bulges and valleys in figure 9*a–c*. In cross-section trajectory image (figure 9*d*), D120C20 has the biggest value of peak-valley difference of three coatings. Its peaks are relatively flat and they reflect bulges. As the content of silica nanoparticles increases, both roughness and peak size on these coatings decrease. The distance between peaks is measured as shown in figure 9*d*. D120C30 has the smallest value and D120C20 has the largest value. Peaks are not regular in D120C20 and D120C30, and slopes of peaks are not measured effectively here. From figure 1*b*, D120C20 has small CA and large SA. Oversize distance between peaks may be the reason for them. D120C25 and D120C30 have similar SA and D120C30 has the largest CA. But D120C30 has a loose coating layer and its smallest bulges are easy abscised, this is the reason for large CA and small SA. The observation from these coatings could be regarded as that increasing filler content would decrease bulge diameter size and diminish distance between peaks. These parameters influencing values of CA and SA effectively can be concluded here.

Wetting state of rough surface is always described with the Wenzle and Cassie–Baxter model. A water droplet is used to be firmly pinned on the surface in the Wenzle state. Composite coatings in this paper have little SA and the water droplet can roll off these surfaces. The Wenzle model does not fit these composite coatings. The Cassie–Baxter model describes a state that water only stays on the surface without intruding in gaps or pits in the surface, and there is air trapped between water and coating surface. Coatings in this paper were fabricated with film former and nanoparticles, water would intrude in the gaps and pits with various depth. Absolute Cassie–Baxter state does not fit these composite coatings. There is a transition state between the Wenzle and Cassie–Baxter states which is suitable for these coatings in this paper [37]. The transition state is described with the Cassie–Baxter model as follows:

$$\cos(\text{CA})=f_{\mathrm{solid}}(\cos(\text{C}{\text{A}}_{E})+1)-1,$$

where CA is the apparent water contact angle, CA_E_ the Yang water contact angle of the smooth surface and *f*_solid_ the solid fraction in solid–air compound surface.

From the Cassie–Baxter model, it is known that the critical factor influencing CA is solid fraction *f*_solid_, and, investigation show that the Cassie–Baxter model could not predict CA exactly [38]. Composite coatings in this paper have irregular shape and the prediction of CA with the Cassie–Baxter model is difficult too, but, diameter size and slope of peaks, distance between peaks on these coatings, may affect the value of *f*_solid_. The relationship between these elements and hydrophobicity of these coatings can be deduced.

**Figure 10.**
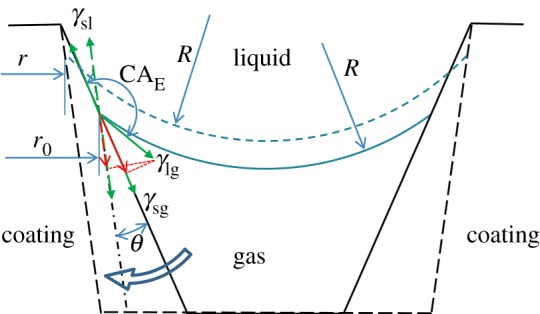
Diagram of effect on CA by slope of peaks.

Figure 10 is a diagram of the effect on CA by slope of peaks. Suppose the coating is in transition state between Wenzle and Cassie–Baxter states. Water surface is in the gap of two peaks. The triple-phase contact line is in balance from the Yang equation. Water surface between two contact areas has to satisfy Laplasse's Law which is listed as follows:

$$\Delta p=\frac{2\gamma_{\mathrm{lg}}}{R},$$

where Δ*p* is pressure difference between inside and outside of the water droplet, *γ*_lg_ the surface tension of the water droplet and *R* the radius of curvature of this water surface.

When peaks change to the dashed line and the slope is the same with the dash-dotted line, the direction of *γ*_sl_ and *γ*_sg_ changes. The component of *γ*_sg_ on the direction of *γ*_sl_ and *γ*_sg_ decreases, and the balance of the triple-phase contact line is broken. The increase of radius of the water surface would break Laplasse's Law. Then, the triple-phase contact line has to be dragged upwards. Both the water surface feeding Laplasse's Law and the triple-phase contact line feeding the Yang equation have to be ensured. The water surface would be pulled to the dashed line with the same radius *R*. The diameter of contact area would be decreased from *r*_0_ to *r*. As a result, the solid fraction *f*_solid_ is diminished and apparent water CA is enlarged. It means that the increasing of peaks slope on the coating surface would increase CA. It should be noted that the peak slope increasing would increase the CA value little for the defects on the triple-phase contact line have a pinning force as the coating is in Cassie–Baxter state. CA of both D60C25 and D120C25 are larger than D15C25 with the former's large slope of peaks in figure 8.

**Figure 11.**
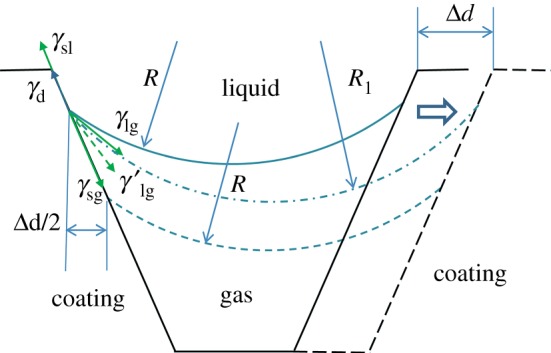
Diagram of effect on CA by distance between peaks.

The distance between peaks is affected with filler content. Larger distance would decrease total contact area between water and coating from the Cassie–Baxter model. But the CA of D120C20 is small, which does not agree with it. The Cassie–Baxter model is ideal without considering the defects on the surface. In figure 11, a diagram of effects on CA by distance between peaks is shown. The triple-phase contact line is under three surface tensions. Water surface between two contact areas has to satisfy Laplasse's Law.

Radius of water surface *R* would be raised as distance increases between two peaks. At this time, pressure difference Δ*p* between inside and outside of the water droplet does not change. The triple-phase contact line would move to another place with the same radius *R* of curvature. The defects on the triple-phase contact line have pinning force *γ*_d_, which will prevent the triple-phase line from moving. Water surface would be balanced in dash-dotted line with radius *R*_1_. Contact areas between coating and water droplet decrease, and solid fraction *f*_solid_ is decreased too. As a result, the apparent CA gets larger from the Cassie–Baxter model. When pinning force *γ*_d_ could not prevent the triple-phase line from moving, the radius of curvature *R* of this water surface would be kept with the original value. At this time, the contact area enlarges and gap trapped air is kept at the original value. As a result, solid fraction *f*_solid_ is enlarged and apparent water CA is decreased. It can be concluded that limited enlarged distance would increase CA, while exceeding enlarged distance would decrease CA. D120C20 in figure 9 having low CA is owing to the large distance of peaks and large diameter size of peaks. On the other side, CA of D60C25 is larger than D15C25 with larger distance size between peaks than that of the latter in figure 8.

**Figure 12.**
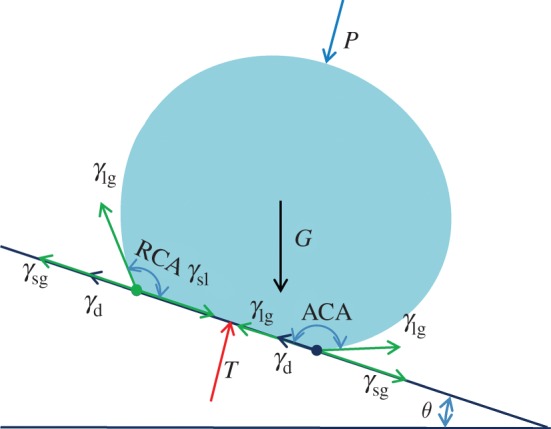
Diagram of force of water droplet on tilt surface.

Though the Cassie–Baxter model described the CA, SA was not explained clearly. SA reflected CA hysteresis [39]. Both chemical heterogeneities and roughness could pin the triple-phase contact line of vapour–liquid–solid, and they were the reason for SA [40]. Forces of the water droplet on a tilt surface are shown in figure 12 and listed as follows:

$$F=G\sin\theta -2\gamma_{d}-\gamma_{\mathrm{lg}}\cos(\text{ACA})+\gamma_{\mathrm{lg}}\cos(\text{RCA}),$$

where *F* is the comprehensive force driving sliding of water droplet, *G*sin*θ* the component of gravity, *γ*_d_ the defects pinning force, *γ*_lg_ the surface tension of water droplet, ACA the approach contact angle and RCA the receding contact angle.

In general, ACA and RCA are close. It can be seen that pinning force acts as critical role for sliding of the water droplet. The observation from the diagram is that pinning force only acts at the triple-phase contact line. The length and the number of defects at the triple-phase contact line are important. Increasing slope of peaks and enlarging the distance between peaks effectively could decrease contact area. The length of the triple-phase contact line decreases too. Both these two means could decrease SA.

**Figure 13.**
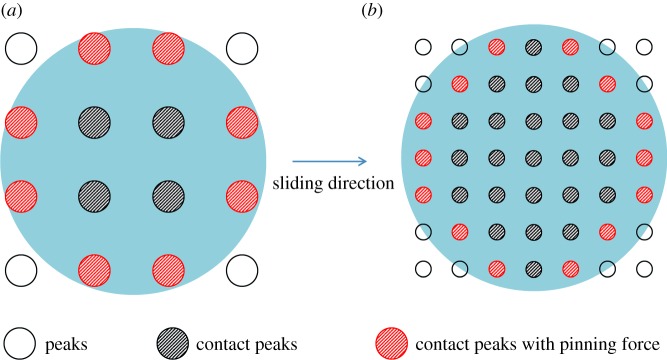
Diagram of contact peaks at two diameters.

Contact area diameter of peaks is complex with effects of shape, distance and slopes of peaks for composite coatings in this paper. It is difficult to decide the CA with this character. The effect of contact area diameter of peaks on SA is investigated. It is supposed that contact areas of composite coatings with different uniform contact area diameters are the same. Large and small diameter peaks are shown in figure 13. The peak diameter in figure 13*a* is twice that in figure 13*b*. The valid length of the triple-phase contact line in figure 13*b* is smaller than that in figure 13*a*. The valid length of the triple-phase contact line with smaller contact area diameters are always less than those with larger contact area diameters. Then, the peak's diameter is positive on decreasing SA value. In figure 8, D120C25 has the smallest contact area diameter, largest slope and appropriate distance between peaks, which has the minimum SA value. In figure 9, D120C30 and D120C25 have similar SA for their similar peak shapes.

From the hydrophobicity measurement result and SEM images, the preferred superhydrophobic composite coating in this paper is D120C25. It has a big slope of peaks, little contact area diameter size and appropriate distance between peaks. All these characters are dominated with dispersion process and filler content. They lead to its large CA and small SA combined with low surface energy of chemical component in these coatings.

### 3.6. Formation mechanism of composite coating based on fluorosilicone resin and modified nanoparticles

It is known that filler content affects coating morphology. In this paper, dispersion process affects surface structure of composite coatings with all modified silica nanoparticles dispersed completely. Smooth, uniform and submicron particles are built on a coating surface with short dispersion time, while irregular larger particles are built on a surface with longer dispersion time. The interactions between nanoparticles and fluorosilicone resin are different under these two conditions. Both fluorosilicone resin and modified silica nanoparticles are inert with a large number of F–C bonds. Compatibility and bonding force between fluorosilicone resin and modified silica particles are weak. Only physical function between them could form a strong interaction.

**Figure 14.**
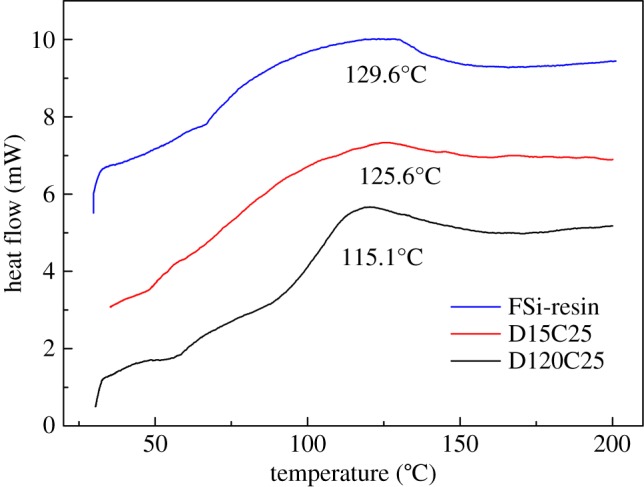
DSC curves of FSi resin, D15C25 and D120C25.

In order to detect the interaction between these two network polymers, DSC analysis was carried out. Figure 14 shows the DSC curves at the heating rate of 20°C min^−1^ of FSi resin, D15C25 and D120C25. FSi resin has the highest glass transition temperature (*T*_g_) at 129.6°C. D15C25 has larger *T*_g_ at 125.6°C than that of FSi resin. D120C25 has the lowest *T*_g_ at 115.1°C. The *T*_g_ difference of three materials proves that the interaction between FSi resin and modified nanoparticles are different. We know curing of fluorosilicone resin is done by cross-linking reaction, and fluorosilicone resin forms network polymer. Besides, investigation showed that PTES could form network polymer for its three hydrolytic functional groups [41]. Two network polymers can form an interpenetrating polymer [42]. *T*_g_ of the interpenetrating polymer would drop as the degree of interpenetrating is enhanced [43,44]. The composite coating in this paper is in good agreement with the interpenetrating polymer model and the formation mechanism of the composite coating can be described as below.

**Figure 15.**
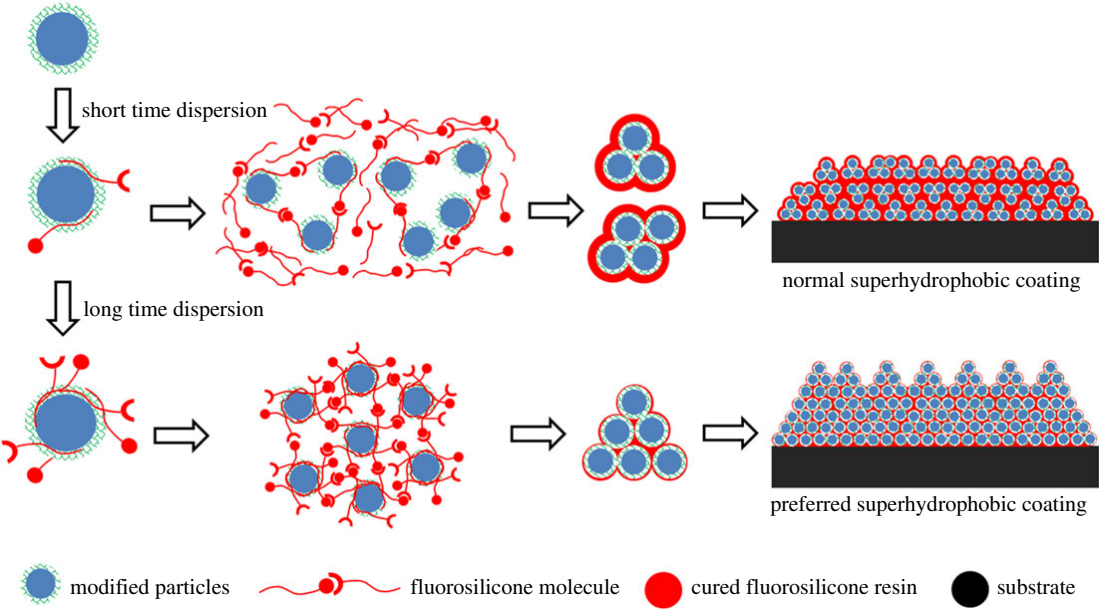
Diagram of formation mechanism of composite coating with superhydrophibicity.

A network polymer layer has been made on the silica nanoparticle surface before the coating is prepared. When modified silica particles are dispersed in solvent, the fluorosilicone molecule would rip into the network polymer of PTES under Brownian movement. The number of fluorosilicone resins ripped into the network polymer is small when dispersion time is short. During curing, interaction between fluorosilicone resin and silica nanoparticles is weak. Silica nanoparticles cannot reunite at large scale and fluorosilicone resin only enwraps these silicone particles. As a result, smooth, uniform and submicron particles are built on a flat coating surface. When extending dispersion time, a large number of fluorosilicone resins rip into the network polymer. Interaction between fluorosilicone resin and nanoparticles are strong. A lot of nanoparticles would reunite to form a large irregular particle. Especially, volatilization of solvent enlarges the gap size of these large particles. Then a rough surface with large irregular particles and big gaps was formed. This rough surface is a typical superhydrophobic one as former sections analysis. The formation mechanism of a flat surface and a rough surface is expressed in figure 15.

## 4. Conclusion

In this paper, we fabricated a composite superhydrophobic coating based on fluorosilicone resin and modified silica nanoparticles. It was made at room temperature without any post-processing. This study concludes that fluorosilicone resin is another preferred polymer with low surface energy to build composite superhydrophobic coating and it has enormous application prospects in superhydrophobic material fabricating. Morphology and topography of composite coatings with different processes were investigated and they have great influence on hydrophobicity. The critical structure factors of superhydrophibic coating were chosen and analysed, which provides directional guidance to build a superhydrophobic surface with other materials and methods. Interaction between inert fluorosilicone resin and modified silica particles with PTES was investigated by analysing morphology and DSC. The interpenetrating polymerization method was used to explain the interaction.

## Data accessibility

This article does not contain any additional data.
